# BRIDGING RESEARCH AND POLICY TO EXPAND SUPPORTS TO OLDER ADULTS LIVING ALONE WITH COGNITIVE IMPAIRMENT IN THE US

**DOI:** 10.1093/geroni/igad104.3789

**Published:** 2023-12-21

**Authors:** Elena Portacolone, Robyn Stone

**Affiliations:** University of California, Berkeley, California, United States; LeadingAge, Washington, District of Columbia, United States

## Abstract

Bridging research and policy allows research findings to have a lasting impact in the wellbeing of vulnerable population such as the estimated 4.3 million older adults living alone with cognitive impairment in the United States. For example, a lasting impact comes from healthcare and social services provided by Medicare and Medicaid tailored to address unmet needs of these populations identified via rigorous research. Despite the importance of bridging research with policy, researchers seldom collaborate with policy makers, thus missing the opportunity of influencing policies. Reasons for these limited collaborations include limited knowledge on methods for researchers to collaborate with policy makers. This session will draw from the expertise gained by the research team of the 5-year Living Alone with Cognitive Impairment Project to involve policy experts in the design of their mixed-method study. Regular interactions between the research team and the Policy Advisory Group led to: 1) a mixed-method design aligned with policy priorities; 2) a report highlighting the priorities of PAG members with regard to policies to enhance the wellbeing of older adults living alone in the US; 3) a series of policy briefs highlighting key findings and related policy recommendations. The oversight of a Policy Advisory Group allowed the Living Alone with Cognitive Impairment Project to focus research activities that are likely to be translated into policy solutions, thus likely to mostly benefit older adults living alone with cognitive impairment, with emphasis on communities of color. These practices can be replicated by researchers in studies supporting underserved populations.

